# Relationships between burnout, turnover intention, job satisfaction, job demands and job resources for mental health personnel in an Australian mental health service

**DOI:** 10.1186/s12913-018-3841-z

**Published:** 2019-01-23

**Authors:** Justin Newton Scanlan, Megan Still

**Affiliations:** 1 Faculty of Health Sciences, The University of Sydney, Room J120, Cumberland Campus C43J, 75 East Street, Lidcombe, NSW Australia; 2 Sydney Local Health District, Mental Health Services, Concord, NSW Australia

**Keywords:** Job demands-resources model, Exhaustion, Disengagement, Employee wellbeing

## Abstract

**Background:**

Burnout and employee turnover in mental health services are costly and can have a negative impact on service user outcomes. Using the Job Demands-Resources model as a foundation, the aim of this study was to explore the relationships between burnout, turnover intention and job satisfaction in relation to specific job demands and job resources present in the workplace in the context of one Australian mental health service with approximately 1100 clinical staff.

**Methods:**

The study took a cross-sectional survey approach. The survey included demographic questions, measures of burnout, turnover intention, job satisfaction, job demands and job resources.

**Results:**

A total of 277 mental health personnel participated. Job satisfaction, turnover intention and burnout were all strongly inter-correlated. The job resources of *rewards and recognition*, *job control*, *feedback* and *participation* were associated with burnout, turnover intention and job satisfaction. Additionally, the job demands of *emotional demands, shiftwork* and *work-home interference* were associated with the exhaustion component of burnout.

**Conclusion:**

This study is the largest of its kind to be completed with Australian mental health personnel. Results can be used as a foundation for the development of strategies designed to reduce burnout and turnover intention and enhance job satisfaction.

**Electronic supplementary material:**

The online version of this article (10.1186/s12913-018-3841-z) contains supplementary material, which is available to authorized users.

## Background

The emotionally demanding nature of mental health work has been proposed to increase the risk of burnout which is also associated with reduced employee satisfaction and higher rates of turnover intention (a desire to leave one’s job) [1–3]. High levels of employee burnout and dissatisfaction are associated with poorer service user outcomes [1, 2, 4, 5] and can have a “contagion” effect to others in the workforce, creating additional difficulties [2, 6]. Additionally, the cost to services of managing high employee turnover is substantial in terms of recruitment, training and loss of organisational knowledge [7–9].

While the consequences of burnout in the mental health workforce have been well-described, the specific features of mental health work that contribute to increased risk of burnout, and other variables of interest such as turnover intention and job satisfaction, have received less attention. Consistent with research in other workforce populations, inter-relationships between burnout, job satisfaction and turnover intention for mental health service personnel have been reported. Burnout and turnover intention are positively correlated and both burnout and turnover intention are negatively associated with job satisfaction [1, 10, 11]. In terms of specific aspects of work, higher levels of burnout have been associated with lower levels of perceived support from colleagues and supervisors, workload pressure, lower levels of perceived autonomy and client-related factors [6, 10–14]. Associations between specific job aspects and turnover intention have been explored less frequently [11, 15], but higher levels of turnover intention have been associated with negative perceptions of management, lower levels of support from supervisors and colleagues, lower levels of autonomy and perceptions of high levels of emotional demands [6, 15–17]. Job satisfaction has also been associated with support from colleagues and supervisors and lower workload pressure [13, 18].

The Job Demands-Resources (JD-R) model of burnout [19–21] proposes that burnout is made up of two primary elements: exhaustion and disengagement. Exhaustion is characterised by the depletion of energy and is the result of enduring physical, affective or cognitive strain. Disengagement is characterised by a distancing of one’s self from one’s work and experiencing negative attitudes towards the work tasks, service recipients or work in general. Additionally, the JD-R proposes that job characteristics can be categorised as either job demands or job resources. Job demands are aspects of work that can cause stress. Job resources are aspects of work that provide support to employees and may help to maintain wellbeing. The original conceptualisation of the JD-R model asserts that high levels of job demands will be associated with higher levels of exhaustion and low levels of job resources will be associated with higher levels of disengagement [21]. Using the JD-R as a framework provides the opportunity for further exploration of the specific job demands and job resources that may be associated with burnout, turnover intention and job satisfaction in the context of the mental health workforce. This has not previously been explored within the mental health workforce.

While a substantial body of work has been published in relation to burnout in the mental health workforce [22], several aspects of the experiences of mental health staff also require further exploration. Examination of the different experiences of different professional groups within mental health services has been limited [1, 15]. Given that most research focuses on one profession or combines various professional groups, there have been calls for more studies that compare the different experiences of professional groups [1]. In addition, there may be differences in experiences of managerial and non-managerial staff and staff working in community or inpatient settings and these are worthy of exploration. Finally, there is a need to further explore the experiences of Australian mental health professionals. While there have been studies exploring the experiences of specific professional groups [23–25], no studies could be located that explored these issues of for the broader mental health workforce in an Australian context. Given the substantial variation in the design of service systems across different countries, findings from other contexts may not apply to the Australian context. This suggests more specific, Australian-based research is necessary.

This study was designed to explore several research hypotheses and questions in an Australian context. The first hypotheses sought to confirm well-established findings from other studies for this population. These hypotheses were: (i) That there will be a positive correlation between burnout and turnover intention and that both will be negatively correlated with job satisfaction; and (ii) That there will be a positive correlation between job demands and the exhaustion component of burnout and a negative correlation between job resources and the disengagement component of burnout. Exploratory research questions were also established: (i) What are the relationships between specific job resources and job demands and burnout, turnover intention and job satisfaction?; (ii) Are there differences in burnout, turnover intention, job satisfaction, job demands or job resources between different groups of mental health staff (professional groups; managerial and non-managerial groups; and community and inpatient staff)? and (iii) What factors (demographic, work-related, job demands and job resources variables) are most strongly related to burnout, turnover intention and job satisfaction?

## Method

### Context

The study was conducted in one large government-funded mental health service in metropolitan Sydney, the state capital of New South Wales, Australia. The mental health service included 21 inpatient units, a large number of community teams spread across 10 service centres and employed approximately 1100 clinical staff.

This study was initiated by the mental health service human resources committee and was approved by a lead Human Research Ethics Committee (overall approval number X11–0162) and approved by three research governance offices responsible for the sites where the research was undertaken.

### Participants

All employees were invited to complete a survey, either online or in pen-and-paper format. Data collection was open for a period of 8 months. Clinical staff came from five primary disciplines: nursing, medical, occupational therapy, psychology and social work.

### Instrumentation

The survey included a range of demographic questions and a suite of scales. Most demographic questions included a “no response” option to ensure that respondents who may have been potentially identifiable through their demographic information had the opportunity to remain anonymous. Elements of the survey relevant to this study are described below. A full copy of the survey is available as Additional file 1.

#### Burnout

Burnout was evaluated using the Oldenburg Burnout Inventory (OLBI) [26, 27]. The OLBI measures two elements of burnout (disengagement and exhaustion), has good internal and external validity and has been used in a variety of industries and across numerous countries, including numerous studies including mental health service personnel (e.g., [14, 25, 28]). Overall scores for disengagement and exhaustion are calculated by averaging responses for items associated with each subscale. Total scores can range from 1 to 4 with higher scores representing higher levels of disengagement or exhaustion. Internal consistency (Cronbach’s α) for the disengagement and exhaustion scales were .75 and .78 respectively.

Although the Maslach Burnout Inventory (MBI) is a more commonly-used measure of burnout, the OLBI was selected for use in this study for numerous reasons. Most importantly, the OLBI is aligned with the JD-R model of burnout. It was developed by the same research team and measures the two key elements of burnout considered in the JD-R, namely disengagement and exhaustion [21]. Secondly, given that it includes both positively- and negatively-worded items, it has been suggested to be psychometrically superior to the MBI [26]. The OLBI has good concurrent validity against the MBI (overall correlations > 0.70), although discriminant validity analyses reveal the OLBI and MBI are independent measures of burnout [26, 29].

#### Turnover intention

Turnover intention was measured by three statements: “I am actively looking for another job,” “As soon as I find another job, I will quit” and “I often think about quitting my job” [30]. Each had three response options: (1) No; (2) Unsure; and (3) Yes. Overall turnover intention was an average of the three responses, with higher scores indicating higher levels of turnover intention. Internal consistency (Cronbach’s α) was .87.

#### Job satisfaction

Overall job satisfaction was rated on a single-item 10-point scale. Participants were asked “Overall, how satisfied are you with your current job?” Response anchors were (1) Very dissatisfied; (5) and (6) Neither satisfied nor dissatisfied; and (10) Very satisfied. While the use of a single item to measure job satisfaction is contentious, previous research has demonstrated the validity of this approach [31–33]. Single-item measures of job satisfaction may be superior to multiple item scales as the single item allows each individual to rate their satisfaction based on job-related factors that are important to them [31, 33].

#### Job demands and job resources

This section included an instrument devised for this study that contained 25 statements. Ten items were related to job demands and 15 were related to job resources. These items were drawn from previous literature [21, 34–36], as no English-language measure of job demands and job resources was available. Areas of job demands covered included: *physical workload*; *time pressure*; *recipient contact demands*; *physical environment*; *shiftwork*; *work-home interference*; *emotional demands*; *workload*; and *cognitive demands*. Job resources included: *feedback*; *rewards and recognition*; *job control*; *participation*; *job security*; *supervisor support*; and *social support*. Items were rated on a five-point Likert scale. All items were coded to demonstrate higher levels of the construct being examined. Items representing the same category were then combined and average scores determined. Internal consistency (Cronbach’s α) for the job demands and job resources scales were .66 and .87 respectively. More information about the job demands and job resources scale is provided in Additional file 2.

### Analyses

All analyses were completed using IBM SPSS Statistics (Version 25, IBM Corporation). Initially descriptive statistics were calculated for demographic variables as well as the research variables included in the study. Then, associations between individual variables were explored to identify the presence of non-linear relationships. Following this, analyses to address each of the research hypotheses and questions were completed.

*Hypothesis 1: That there will be a positive correlation between burnout and turnover intention and that both will be negatively correlated with job satisfaction*. Bivariate correlations (Pearson’s product moment correlation coefficients) were calculated for relationships between disengagement and exhaustion (the two components of burnout), turnover intention and job satisfaction.

*Hypothesis 2: That there will be a positive correlation between job demands and the exhaustion component of burnout and a negative correlation between job resources and the disengagement component of burnout*. Correlation coefficients were calculated for relationships between job demands and exhaustion and job resources and disengagement.

*Research question 1: What are the relationships between specific job resources and job demands and burnout, turnover intention and job satisfaction?* Correlation coefficients were calculated to explore these relationships.

*Research question 2: Are there differences in burnout, turnover intention, job satisfaction, job demands or job resources between different groups of mental health staff (professional groups; managerial and non-managerial groups; and community and inpatient staff)?* For between-group analyses across professional groups, the dataset was firstly restricted to respondents from the five main disciplines within the mental health service (i.e., medical, nursing, occupational therapy, psychology and social work). Following this, a series of analyses of variance were used to explore for differences between these groups in terms of disengagement, exhaustion, turnover intention and job satisfaction. All respondents were classified as manager or non-manager based on their responses to questions about job classification and work role. Managers’ and non-managers’ means for disengagement, exhaustion, turnover intention and job satisfaction were then compared using independent-samples *t*-tests. Finally, the sample was restricted to those individuals who reported working in inpatient or community settings and comparisons between inpatient- and community-based staff were made using independent-samples *t*-tests.

*Research question 3: What factors (demographic, work related, job demands and job resources variables) are most strongly related to burnout, turnover intention and job satisfaction.* The final set of analyses involved the development of linear stepwise regression models for each of the main variables of interest (disengagement, exhaustion, turnover intention and job satisfaction). Each model included one of the main variables as the dependent variable and independent variables included demographic variables (age and gender), work-related variables and categories of job demands and job resources. Work-related variables included: profession type (each profession was included as a dummy variable), manager or non-manager role, community or inpatient setting, self-classification of how “generic” or “profession specific” the role was, full-time or part-time status (and whether this aligned with their working hours preference) and years of experience in mental health services.

## Results

### Respondents

A total of 277 managers and clinicians (approximately 25% response rate) completed surveys. Demographics of the sample are summarised in Table 1. Approximately two-thirds of the sample was female. Nurses made up almost half of the sample with approximately even representation of the four other major professional groups, which was broadly reflective of the overall makeup of the mental health service’s workforce. Respondents were also relatively evenly spread in terms of working in inpatient or community settings, age and years of experience working in mental health.

**Table 1:** Demographics of the sample

Domain	Characteristic	Frequency	Percent
Gender	Female	178	64.3%
	Male	91	32.9%
	Not stated	8	2.9%
Age	30 or under	47	17.0%
	31–40	78	28.2%
	41–50	59	21.3%
	Over 50	73	26.4%
	Not stated	20	7.2%
Discipline	Medical	43	15.5%
	Nursing	123	44.4%
	Occupational Therapy	34	12.3%
	Psychology	32	11.6%
	Social Work	26	9.4%
	Other	4	1.4%
	Not stated	15	5.4%
Managerial status	Manager	29	10.5%
	Non-manager	248	89.5%
Main work location	Inpatient	127	45.8%
	Community	129	46.6%
	Other	16	5.8%
	Not stated	5	1.8%
Generic vs profession specific role	Mainly generic	25	9.0%
	More generic than specific	45	16.2%
	About half-half	52	18.8%
	More specific than generic	62	22.4%
	Mainly specific	81	29.2%
	Unsure / not applicable	12	4.3%
Length of time working in mental health	Less than 1 year	8	2.9%
	1–2 years	26	9.4%
	2–5 years	56	20.2%
	5–10 years	66	23.8%
	10–20 years	61	22.0%
	Over 20 years	60	21.7%

### Descriptive statistics

Descriptive statistics for key variables are presented in Table 2. For burnout, mean scores for disengagement and exhaustion were 2.24 and 2.38, respectively (on a scale of 1 to 4). Turnover intention was low with a mean rating of 1.5 (on a scale of 1 to 3). Mean job satisfaction was 6.9 on the 10-point scale. Mean ratings for job demands and job resources were 3.38 and 3.69, respectively (on a scale of 1 to 5). Mean scores for specific types of job demands and job resources are also presented in Table 2. Exploration of the associations between variables revealed that there were no obvious non-linear relationships present.

**Table 2:** Descriptive and correlation statistics for key variables

Component	Potential Range	Mean (S.D.)	Di0073	Correlation with		
				Exh	T.I.	Sat.
Disengagement	1 to 4	2.24 (0.40)	–	.56^***^	.55^***^	−.57^***^
Exhaustion	1 to 4	2.38 (0.41)	.56^***^	–	.42^***^	−.44^***^
Turnover Intention	1 to 3	1.46 (0.66)	.55^***^	.42^***^	–	−.50^***^
Job satisfaction	1 to 10	6.94 (2.04)	−.57^***^	−.44^***^	−.50^***^	–
Job Demands	1 to 5	3.38 (0.46)	.33^***^	.51^***^	.22^***^	−.18^**^
Physical Workload	1 to 5	2.79 (0.98)	.19^**^	.20^***^	.16^**^	−.14^*^
Recipient Contact Demands	1 to 5	3.88 (0.77)	.09	.27^***^	.12^*^	−.09
Physical Environment	1 to 5	2.87 (1.13)	.24^***^	.28^***^	.18^**^	−.14^*^
Shiftwork	1 to 5	2.32 (0.93)	.27^***^	.27^***^	.11	−.10
Work-Home Interference	1 to 5	2.34 (1.04)	.27^***^	.40^***^	.19^**^	−.22^***^
Emotional Demands	1 to 5	4.05 (0.84)	.08	.32^***^	.09	−.05
Time Pressure	1 to 5	3.32 (1.13)	.28^***^	.32^***^	.11	−.18^**^
Workload	1 to 5	3.43 (0.93)	.12^*^	.25^***^	.10	−.13^*^
Cognitive Demands	1 to 5	4.38 (0.67)	−.02	.07	.00	.12^*^
Job Resources	1 to 5	3.69 (0.53)	−.50^***^	−.36^***^	−.42^***^	.46^***^
Feedback	1 to 5	3.35 (0.91)	−.36^***^	−.29^***^	−.27^***^	.35^***^
Rewards and recognition	1 to 5	3.38 (0.76)	−.46^**^	−.34^***^	−.36^**^	.41^**^
Job Control	1 to 5	4.00 (0.68)	−.40^***^	−.28^***^	−.33^***^	.38^***^
Participation	1 to 5	3.42 (0.95)	−.29^***^	−.20^***^	−.22^***^	.24^***^
Job Security	1 to 5	3.97 (0.85)	−.10	−.03	−.08	.06
Supervisor Support	1 to 5	3.56 (0.84)	−.29^***^	−.18^**^	−.31^***^	.28^***^
Social Support	1 to 5	4.20 (0.60)	−.36^***^	−.27^***^	−.26^***^	.31^***^

*Dis* Disengagement; *Exh* Exhaustion; *T.I.* Turnover intention; *Sat* Job satisfaction

Notes: ^*^ *p* < .05, ^**^ *p* < .01, ^***^ *p* < .001

### Hypothesis 1:

*That there will be a positive correlation between burnout and turnover intention and that both will be negatively correlated with job satisfaction*.

Bivariate correlations are presented in Table 2. This hypothesis was supported. The correlation between disengagement and turnover intention (*r* = 0.55, *p* < .001) was stronger than the correlation between exhaustion and turnover intention (*r* = 0.42, *p* < .001; *z* = 2.04, *p* = 0.04). Both elements of burnout (disengagement and exhaustion) and turnover intention were negatively correlated with job satisfaction.

### Hypothesis 2:

*That there will be a positive correlation between job demands and the exhaustion component of burnout and a negative correlation between job resources and the disengagement component of burnout.*

Correlations between these elements are also presented in Table 2. This hypothesis was also supported. There was a positive correlation between job demands and exhaustion (*r* = 0.51, *p* < .001) and a negative correlation between job resources and disengagement (*r* = − 0.50, *p* < .001).

### Research question 1:

*What are the relationships between specific job resources and job demands and burnout, turnover intention and job satisfaction?*

Correlations between specific job demands and job resources and burnout, turnover intention and job satisfaction are also presented in Table 2. Except for *cognitive demands*, all job demands had a positive correlation with exhaustion. Amongst job demands, *cognitive demands* had the most unusual pattern of associations. There was no relationship between *cognitive demands* and disengagement, exhaustion or turnover intention and there was a positive relationship between *cognitive demands* and job satisfaction (i.e., those who reported higher levels of *cognitive demands* were more likely to report higher levels of job satisfaction). Except for *job security*, all job resources had the same pattern of relationships with the key study variables: negative relationships with disengagement, exhaustion and turnover intention and positive relationships with job satisfaction.

### Research question 2:

*Are there differences in burnout, turnover intention, job satisfaction, job demands or job resources between different groups of mental health staff (professional groups; managerial and non-managerial groups; and community and inpatient staff)?*

Descriptive data and results from the ANOVA and *t*-tests exploring between-group differences are presented in Table 3. Apart from managers having slightly higher job satisfaction and slightly lower turnover intention than non-managers, no other between-group differences were detected.

**Table 3:** Descriptive statistics and between-group analyses for key variables according to different workforce sub-groups

Workforce sub-group	Disengagement	Exhaustion	Turnover Intention	Job Satisfaction	Demands	Resources
	Mean (SD)	Mean (SD)	Mean (SD)	Mean (SD)	Mean (SD)	Mean (SD)
All participants (n = 277)	2.24 (0.40)	2.38 (0.41)	1.46 (0.66)	6.94 (2.04)	3.38 (0.46)	3.69 (0.53)
Professional group						
Medical (n = 43)	2.10 (0.43)	2.24 (0.42)	1.23 (0.48)	7.32 (1.94)	3.39 (0.55)	3.73 (0.46)
Nursing (n = 123)	2.28 (0.38)	2.35 (0.41)	1.48 (0.69)	6.96 (2.02)	3.38 (0.50)	3.68 (0.55)
Occupational Therapy (n = 34)	2.17 (0.42)	2.41 (0.46)	1.57 (0.73)	7.21 (2.07)	3.31 (0.42)	3.82 (0.51)
Psychology (n = 32)	2.20 (0.39)	2.42 (0.42)	1.26 (0.45)	6.97 (2.31)	3.42 (0.36)	3.81 (0.49)
Social Work (n = 26)	2.30 (0.31)	2.47 (0.31)	1.46 (0.61)	6.64 (1.80)	3.47 (0.41)	3.61 (0.53)
Between groups differences (ANOVA, F value)	*0.06*	*0.14*	*0.07*	*0.70*	*0.78*	*0.37*
Management role						
Manager (n = 29)	2.12 (0.37)	2.33 (0.39)	1.23 (0.51)	7.66 (1.70)	3.43 (0.46)	3.77 (0.46)
Not manager (n = 248)	2.25 (0.40)	2.38 (0.42)	1.49 (0.68)	6.86 (2.06)	3.37 (0.47)	3.68 (0.54)
Between groups differences (t-test, t-value)	*−1.63*	*−0.62*	−2.50*	1.99*	*0.62*	*0.85*
Work setting						
Inpatient (n = 127)	2.24 (0.38)	2.34 (0.38)	1.44 (0.64)	7.18 (1.86)	3.35 (0.47)	3.71 (0.53)
Community (n = 129)	2.25 (0.43)	2.44 (0.44)	1.49 (0.69)	6.71 (2.14)	3.42 (0.47)	3.67 (0.54)
Between groups differences (t-test, t-value)	*−0.22*	*−1.87*	*− 0.55*	*1.86*	*− 1.22*	*0.53*

Note: * *p* < 0.05, ** *p* < .01, *** *p* < .001

### Research question 3:

*What factors (demographic, work related, job demands and job resources variables) are most strongly related to burnout, turnover intention and job satisfaction.*

Results from the regression analyses are presented in Table 4. The job resource of *rewards and recognition* was the strongest predictor of variance in disengagement and turnover intention (predicting 20 and 14% of the variance, respectively). The job demand of *emotional demands* was the strongest predictor of variance in exhaustion, predicting 16% of the variance. Finally, the job resource of *feedback* was the strongest predictor of variance in job satisfaction, predicting 11% of the variance.

**Table 4:** Results from stepwise linear regression analyses

Model	Beta^a^	Adj. *R*^*2*^ change	*F* value	*P* value
Dependent variable: Disengagement				
Rewards and recognition	−.30	.20		
Shiftwork	.20	.05		
Time Pressure	.17	.03		
Medical staff member	−.17	.02		
Job Control	−.17	.02		
Overall model		.32	20.62	< .001
Dependent variable: Exhaustion				
Emotional demands	.25	.16		
Shiftwork	.21	.07		
Feedback	−.16	.05		
Work-Home interference	.17	.03		
Age	−.19	.04		
Time pressure	.14	.02		
Male gender	−.14	.01		
Physical environment	.15	.02		
Working in community setting	.11	.01		
Overall model		.41	16.87	< .001
Dependent variable: Turnover intention				
Rewards and recognition	−.24	.14		
Job Control	−.24	.03		
Recipient contact demands	.19	.02		
Medical staff member	−.13	.02		
Overall model		.21	14.42	< .001
Dependent variable: Job satisfaction				
Feedback	.22	.11		
Participation	.18	.05		
Medical staff member	.17	.02		
Job Control	.17	.01		
Recipient contact demands	−.13	.01		
Overall model		.20	11.45	< .001

Adj. *R*^*2*^ = Adjusted *R*^*2*^

^a^ Standardised coefficient for variable in final model

## Discussion

This study extends the current knowledge of the workforce experiences of mental health workers in Australia. To our knowledge, this is the largest study to be undertaken in the Australian mental health context. Additionally, this study provides analyses of specific job demands and resources and their associations with a variety of outcomes for workers as well as exploration of differences present between various staff groupings.

As expected, there were significant relationships between all of the key variables: burnout, turnover intention and job satisfaction. This is consistent with previous research [37–40]. Similarly there were significant relationships between job demands and exhaustion and inverse relationships between most job resources and disengagement. These results provide further support to the increasing evidence base for usefulness of the Job Demands-Resources model of burnout in understanding workplace experiences [19, 20]. The unusual bivariate relationships between *cognitive demands* and *job security* and the key study variables are worthy of further exploration. Unlike all other job demands explored, there was no relationship between *cognitive demands* and exhaustion. Similarly there were no relationships between *cognitive demands* and disengagement or turnover intention. Perhaps most interestingly, there was a weak, but positive relationship between *cognitive demands* and job satisfaction. The direction of this relationship is different to all of the other job demands explored in this study. Van den Broeck et al. [35] suggested that job demands could be further classified into job hindrances and job challenges. They suggested that while job hindrances would consistently have a negative impact, job challenges may, in certain circumstances, have a positive impact on workers. Results from this study suggest that such a relationship may be present for *cognitive demands*: that those individuals who find their work cognitively challenging may be more satisfied with their jobs. Previous research on precarious work has highlighted the very deleterious effects of poor job security [41, 42], so the lack of relationship between *job security* and the main study variables is surprising. However, in the context of this study, the vast majority (80.1%) of participants agreed or strongly agreed that “my job is secure”, which is likely to have influenced these results. This suggests that while lack of job security may be associated with negative outcomes, the converse: that the presence of job security would be associated with positive outcomes; may not be true.

This study has also added to the knowledge of differences in experiences of individuals within different professional groups within mental health services. In the overall analyses, no between-group differences were found between professional groups in any of the key variables. This finding is in contrast to findings from Onyett and colleagues [43] who reported significant differences in burnout and job satisfaction between different staff groups in community mental health, but consistent with the finding from Yanchus and colleagues [44] who reported that there were no differences between professional groups in terms of turnover intention. It should be noted, however, that the dummy variable “medical staff member” did predict a small amount of variance in the regression analyses for disengagement, turnover intention and job satisfaction. On average medical staff members’ ratings for disengagement and turnover intention were numerically lower than for staff from other professionals groups and their ratings for satisfaction were numerically higher than for staff from other professional groups.

In the Australian context, it has been argued that changes to more community-based service provision and the widespread adoption of “generic” roles such as case management in community services could increase the risk of burnout and job dissatisfaction [24]. The comparison of inpatient settings (where roles tend to be more “profession specific”) and community settings (where roles tend to be more “generic”) in this study enabled the examination of this hypothesis. The finding that there were no differences between inpatient and community staff on any of the variables suggests that, at least for this group of participants, those differences were not associated with poorer outcomes for community-based staff.

The lower level of turnover intention and higher job satisfaction reported by managers in comparison to non-managers is noteworthy, but difficult to interpret. While some literature suggests that organisational structures that support managers’ autonomy and flexibility, manageable workloads and limited work-life interference are associated with job satisfaction lower turnover intention [45, 46], if this were the case, then it would be seen in terms of higher levels of job resources, which was not the case in this study. Therefore other factors, such as organisational commitment, intrinsic work motivations or core self-evaluation [47–49], not measured in this study may explain these differences.

Results from the regression analyses also provide useful information in terms of better understanding the experiences of mental health personnel in Australia. Consistent with the theory underpinning the Job Demands-Resources model of burnout [20, 21], job resources were most influential in terms of predicting disengagement. However, the job demands of *shiftwork* and *time pressure* also exerted a significant influence. Similarly, while a range of job demands were most influential in the prediction of variance in exhaustion, the job resource of *feedback* was also influential. An increasing number of studies have explored the potential “buffering” effect of job resources against the negative impact of job demands [20]. The potentially attenuating role of the job resource of *feedback* found in the current study is consistent with findings from previous studies [34, 50]. An additional finding from the regression analyses is that, in comparison to job demands, job resources appear more influential in supporting higher job satisfaction and lower turnover intention. This is further supported by the stronger correlations seen between overall job resources in comparison to job demands and job satisfaction (0.46 cf. -0.18, *z* = 3.69, *p* < 0.001) and turnover intention (− 0.42 cf. 0.22, *z* = 2.62, *p* = 0.004).

Overall, in the context of work in mental health, job resources are likely to be more amenable to being improved in contrast to job demands being reduced. In terms of job resources, *rewards and recognition* and *job control* contributed to lower levels of disengagement and turnover intention. *Feedback* contributed to lower levels of exhaustion and higher job satisfaction. *Participation* and *job control* also contributed to higher levels of job satisfaction. Participants reported quite high levels of *job control* (mean of 4.00 on the 5-point scale), but lower levels of *feedback*, *rewards and recognition* and *participation* (means of 3.35, 3.38 and 3.42 respectively). This suggests that a focus on improving *feedback*, *rewards and recognition* and *participation* may result in positive outcomes. Previous literature has demonstrated that changes in leadership style towards giving regular feedback and recognising staff achievements in both public and private contexts is associated with higher levels of job satisfaction and lower levels of burnout [51–53]. Additionally, inclusive and consultative leadership styles (referred to as “high leader-member exchange” styles) have been reported to improve employees’ job satisfaction and reduce burnout [54].

In terms of job demands, the job demand most associated with exhaustion was *emotional demands* and *recipient contact demands* had a small but significant influence on job satisfaction and turnover intention. These job demands were reported frequently (means of 4.05 and 3.88 respectively on the 5-point scale). While these job demands are typically considered a core aspect of mental health service delivery, previous research has indicated that training and support for recovery-oriented practice can lead to an increase in therapeutic optimism [55]. This may go some way to addressing the emotionally challenging aspects of work in mental health. Other influential job demands were *shiftwork*, *work-home interference* and *time pressure*. While *time pressure* (related to workload) is challenging to address in the context of limited resources, individualised flexible work arrangements, where practical, may go some way to reducing the impact of *shiftwork* and *work-home interference* [56].

### Study considerations

Although this study was, to our knowledge, the largest Australian study of its type involving the multidisciplinary workforce in mental health, there are some limitations that should be noted. First, the sample was not random and the response rate (approximately 25%) was relatively low. Therefore, respondents may not be reflective of the wider mental health workforce. It could be that employees with more positive or more negative experiences may have been more likely to participate in the study as opposed to those employees with less extreme workforce experiences. Alternatively, those who were most burnt out may have been less likely to respond. Second, the measure of job demands and resources was designed for this study only and has not been formally evaluated. The internal consistency for the job demands scale was quite low, so interpretations of results using this scale should be made with caution. Third, all measures were self-report. The absence of objective measures of burnout or job characteristics is common to much research in this area, but does mean that individuals’ interpretations of their circumstances may vary and this may influence the overall results of this study. Additionally, the fact that participants for this study were drawn from only one mental health service means that results from this study may not be generalisable to staff from other mental health services. Finally, and perhaps most importantly, the data used in this study was cross-sectional only. This means that although relationships can be explored, it is not possible to infer causal directions between different variables. While it is hypothesised that varying levels of job demands and job resources lead to varying levels of burnout, the opposite may also be true. Those individuals who experience higher levels of burnout may perceive their work as more demanding and may perceive supports to be lower than employees who experience lower levels of burnout.

## Conclusion

This study is the largest of its kind to be completed with a multidisciplinary sample of Australian mental health professionals. Consistent with previous research, job satisfaction, turnover intention and burnout were all strongly inter-correlated. The job resources of *rewards and recognition*, *job control*, *feedback* and *participation* were most strongly associated with lower levels of burnout, lower turnover intention and higher job satisfaction. The job demands of *emotional demands, shiftwork* and *work-home interference* were associated with higher levels of exhaustion.

This study has highlighted some potential key correlates of both positive and negative employee outcomes in the context of one large mental health service in Australia. Future research should replicate this study with groups of mental health clinicians to determine whether the results found here are applicable to the overall population of mental health clinicians. Additionally, future research should attempt to explore the causal direction of the relationships between job demands and resources and employee outcomes through the use of longitudinal designs as well as exploring the effectiveness of strategies designed to reduce burnout and turnover intention and improve job satisfaction.

## Additional files

Additional file 1: Workforce Survey Questionnaire. Full survey used as the data collection tool in this study. (PDF 392 kb)

Additional file 2: Job Demands Resources Questionnaire information. Further information on the development of the Job Demands Resources Questionnaire used in this study. (PDF 103 kb)

## Abbreviations

JD-R: Job Demands-Resources model of burnout
MBI: Maslach Burnout Inventory
OBLI: Oldenburg Burnout Inventory
