# Supplementary material for: Relationships between burnout, turnover intention, job satisfaction, job demands and job resources for mental health personnel in an Australian mental health service
Source: BMC Health Serv Res. 2019 Jan 23;19:62. doi: 10.1186/s12913-018-3841-z (PMC6343271; doi:10.1186/s12913-018-3841-z)
Supplement: Supplementary file 1 — Workforce Survey Questionnaire. Full survey used as the data collection tool in this study. (PDF 392 kb) [file 12913_2018_3841_MOESM1_ESM.pdf]

MENTAL HEALTH SERVICE WORKFORCE SURVEY

This survey is designed to explore issues related to recruitment and retention and factors related to workforce satisfaction and burnout in the Mental Health Service. Your responses will assist the Mental Health Service to implement strategies aimed towards making the Mental Health Service a better place to work.

This questionnaire will take approximately 15 minutes to complete.

Note: Your confidentiality in completing this survey is assured. Whilst we have no intention of identifying individuals, some individuals may be identifiable through their responses (e.g., if you are the only social worker on a particular team). If this is the case, you may choose to select the response option "I'd rather not say." We do ask, however, that you provide as much information as possible to allow us to complete analyses comparing different groups (to more effectively target the strategies we implement).

Your participation in this project is entirely voluntary. Your choice to participate or not will have no impact on your relationships with others in the organisation. This project has been approved by the SLHN Human Research Ethics Committee – CRGH and the SLHN Human Research Ethics Committee – RPAH Zone.

What is your current MAIN work team (e.g., Manning Unit, Gna Ka Lun, Bankstown Community Rehab; Canterbury Early Intervention in Psychosis; etc).

(please enter your response here) ➔	

☐ I'd rather not say

What is your discipline?

- ☐ Medical
- ☐ Nursing
- ☐ Occ. Therapy
- ☐ Psychology
- ☐ Social work
- ☐ Administration
- ☐ Management
- ☐ Other (please specify) ________________
- ☐ I'd rather not say

What is your current level / grade (e.g., Registered Nurse, Clinical Nurse Specialist, Enrolled Nurse, Level 2 Occupational Therapist, Clinical Psychologist, Admin Officer Grade 2, Health Service Manager Level 2, etc)?

(please enter your response here) ➔	

☐ I'd rather not say

What is your MAIN current role?

- ☐ Management
- ☐ Administration / Support
- ☐ Inpatient adolescent (ICAMHS)
- ☐ Inpatient SMHSOP
- ☐ Inpatient high dependency / IPCU / observation unit
- ☐ Inpatient general adult acute
- ☐ Inpatient rehabilitation
- ☐ Community acute care / CoMHET
- ☐ Community case management
- ☐ Community rehabilitation
- ☐ Community ICAMHS
- ☐ Community early intervention / early psychosis
- ☐ Community SMHSOP
- ☐ Other (please specify) ____________________
- ☐ I'd rather not say

Would you say that your current MAIN position is:
(NB: In this question, 'generic' refers to positions where roles and responsibilies are performed by a range of professionals - for example case management, care coordination or Acute Care Teams / CoMHET; whereas 'discipline-specific' refers to roles / duties that are specific to the discipline group - for example, inpatient social worker; rehabilitation occupational therapist; clinical nurse specialist)

- ☐ Totally / almost totally discipline specific
- ☐ More discipline-specific than generic
- ☐ About half discipline specific and half generic
- ☐ More generic than discipline specific
- ☐ Totally/ almost totally generic
- ☐ Not applicable

In terms of your working hours (and your work needs / preferences), please select the most appropriate response:

- ☐ I am full time and this generally meets my needs
- ☐ I am full time but would prefer / need to work part time
- ☐ I am part-time and this generally meets my needs
- ☐ I am part-time but would prefer / need to work full time
- ☐ I am casual and this generally meets my needs
- ☐ I am casual but would prefer / need to work part-time
- ☐ I am casual but would prefer / need to work full-time
- ☐ I'd rather not say
- ☐ Other (please specify)____

What is your gender?

- ☐ Female
- ☐ Male
- ☐ I'd rather not say

What is your age?

☐ Under 30 ☐ 30-40 ☐ 40-50 ☐ Over 50
☐ I'd rather not say

How long have you been working for SSWAHS / Sydney LHN or South Western Sydney LHN?

☐ Less than 1 year ☐ 1-2 years ☐ 2-5 years
☐ 5-10 years ☐ 10-20 years ☐ Over 20 years

In total, how long have you worked in mental health?

☐ Less than 1 year ☐ 1-2 years ☐ 2-5 years
☐ 5-10 years ☐ 10-20 years ☐ Over 20 years

On a scale of 1 to 10, how satisfied are you with your current job? (Circle the most appropriate number)

	Very dissatisfied				Neither satisfied nor dissatisfied					Very satisfied
**circle your response**	**1**	**2**	**3**	**4**	**5**	**6**	**7**	**8**	**9**	**10**

I applied for my current position because…

	Not a particular attraction / Not applicable	Somewhat of an attraction	Quite an important attraction
…the organisation has a good reputation	☐	☐	☐
…I'd done a student placement here	☐	☐	☐
…the education and training programs were good	☐	☐	☐
…I knew other people who worked here	☐	☐	☐
…I was interested in the opportunities to develop more skills	☐	☐	☐
…I was really interested in the clinical role / type of work involved in the role	☐	☐	☐
…the salary was good	☐	☐	☐
…I'd heard that the particular team was good	☐	☐	☐
…I was just applying for whatever jobs were available	☐	☐	☐
…the service / team has uses evidence based / best practice methods	☐	☐	☐
…the opportunity for involvement in quality improvement / research	☐	☐	☐
…I thought the service offered good career opportunities	☐	☐	☐
…I'd worked for the organisation before and felt comfortable here	☐	☐	☐
…the organisation / team appeared dynamic and / or had a strong vision	☐	☐	☐
…I was interested in working in mental health	☐	☐	☐
…I thought the position would offer me the opportunity for flexibility (e.g., working hours, working days, study leave)	☐	☐	☐
…there appeared to be a lot of variety in the role	☐	☐	☐
…the location was good	☐	☐	☐
…the resources and infrastructure available were good	☐	☐	☐
…there were lots of opportunities for overtime and / or shiftwork	☐	☐	☐
…my working visa was sponsored by the organisation	☐	☐	☐
…it was helpful in securing my visa / residency status	☐	☐	☐
Other (please list)			

Please list any factors that you like about your current position that keep you coming back to work each day.

Please list any factors that you do not like about your current position.

What factors have led you to leave jobs in the past or to seriously consider leaving your current position?

The next questions are related to whether you are currently considering leaving the organisation.

	Yes	*Unsure*	*No*
I am actively looking for another job	☐	☐	☐
As soon as I can find another job, I will quit	☐	☐	☐
I often think about quitting my job	☐	☐	☐
If you are considering quitting, what are the main reasons for this?			

The next questions are related to your perception of a variety of characteristics of your current position

	Strongly disagree	*Disagree*	*Neutral*	*Agree*	*Strongly Agree*
My achievements are recognised by my supervisor / manager	☐	☐	☐	☐	☐
The job requires me to keep track of more than one thing at a time	☐	☐	☐	☐	☐
My work requires a lot of concentration	☐	☐	☐	☐	☐
The job gives me a chance to use my personal initiative or judgment in carrying out the work	☐	☐	☐	☐	☐
Only the management decides what everybody has to do	☐	☐	☐	☐	☐
My work is emotionally demanding	☐	☐	☐	☐	☐
My supervisor is concerned about the welfare of the people that work for him/her	☐	☐	☐	☐	☐
The job gives me considerable opportunity for independence and freedom in how I do the work	☐	☐	☐	☐	☐
I always have enough time to perform my tasks	☐	☐	☐	☐	☐
I receive feedback on my performance from other people in my organization (such as my manager or coworkers)	☐	☐	☐	☐	☐
I get enough feedback about the quality of my performance	☐	☐	☐	☐	☐
My supervisor / manager inspires me to do my best work	☐	☐	☐	☐	☐
My colleagues are willing to give me help if I ask for it	☐	☐	☐	☐	☐
My job is physically taxing	☐	☐	☐	☐	☐
It is physically taxing for me to get used to my working times	☐	☐	☐	☐	☐
My achievements are recognised by my colleagues	☐	☐	☐	☐	☐
My performance is rewarded properly	☐	☐	☐	☐	☐
My supervisor uses his / her influence to help me solve problems	☐	☐	☐	☐	☐
The job allows me to make my own decisions about how to schedule my work	☐	☐	☐	☐	☐
My working hours allow me to meet my family and personal obligations	☐	☐	☐	☐	☐
My physical working conditions – climate, light, noise, design of the working place, and material – are alright	☐	☐	☐	☐	☐
My job requires working very hard or very fast	☐	☐	☐	☐	☐
I feel my job is secure	☐	☐	☐	☐	☐
My contact with persons to whom I have to offer services is demanding	☐	☐	☐	☐	☐
I have good relationships with my colleagues	☐	☐	☐	☐	☐

The final set of questions is related to your feelings about your current work.

	Strongly disagree	*Disagree*	*Agree*	*Strongly agree*
I always find new and interesting aspects in my work.	☐	☐	☐	☐
There are days when I feel tired before I arrive at work.	☐	☐	☐	☐
It happens more and more often that I talk about my work in a negative way.	☐	☐	☐	☐
After work, I tend to need more time than in the past in order to relax and feel better.	☐	☐	☐	☐
I can tolerate the pressure of my work very well.	☐	☐	☐	☐
Lately, I tend to think less at work and do my job almost mechanically.	☐	☐	☐	☐
I find my work to be a positive challenge.	☐	☐	☐	☐
During my work, I often feel emotionally drained.	☐	☐	☐	☐
Over time, one can become disconnected from this type of work.	☐	☐	☐	☐
After working, I have enough energy for my leisure activities.	☐	☐	☐	☐
Sometimes I feel sickened by my work tasks.	☐	☐	☐	☐
After my work, I usually feel worn out and weary.	☐	☐	☐	☐
This is the only type of work that I can imagine myself doing.	☐	☐	☐	☐
Usually, I can manage the amount of my work well.	☐	☐	☐	☐
I feel more and more engaged in my work.	☐	☐	☐	☐
When I work, I usually feel energized.	☐	☐	☐	☐

If completing this questionnaire has raised any issues which are concerning or that you would like to discuss, you can access free, professional and strictly confidential counselling and support services through the Employee Assistance Program:

Balmain	9515 9681	Campbelltown	4634 3591	Fairfield	9828 4605
Bankstown	9722 8230	Canterbury	9787 0244	Liverpool	9828 4552
Bowral	9828 4552	Concord	9767 7053	RPA (Infoline)	9515 9688

Thank you for completing this survey.

Please return it in the attached envelope to:

Workforce Survey, Human Resources Committee,
c/- Lynne Robinson, Level 1 Administration, Concord Centre for Mental Health,
CRGH, Hospital Road, Concord, 2139
