# Supplementary material for: Relationships between burnout, turnover intention, job satisfaction, job demands and job resources for mental health personnel in an Australian mental health service
Source: BMC Health Serv Res. 2019 Jan 23;19:62. doi: 10.1186/s12913-018-3841-z (PMC6343271; doi:10.1186/s12913-018-3841-z)
Supplement: Supplementary file 2 — Job Demands Resources Questionnaire information. Further information on the development of the Job Demands Resources Questionnaire used in this study. (PDF 103 kb) [file 12913_2018_3841_MOESM2_ESM.pdf]

SUPPLEMENTARY ONLINE MATERIAL

Job Demands Resources Questionnaire

Introduction

As no questionnaires exploring job demands and job resources were available in English, the Job Demands Resources Questionnaire was developed from items and concepts reported in published literature.

Within the questionnaire, there are 10 statements referring to job demands and 15 statements referring to job resources. These are then further broken down into sub-categories to allow for the exploration of relationships between specific job demands or job resources and various outcome variables (e.g., burnout, turnover intention or job satisfaction).

Respondents are requested to report their level of agreement with each statement according to a 5-piont Likert scale (1 = strongly disagree, 2 = disagree, 3 = neither agree nor disagree; 4 = agree, 5 = strongly agree).

Items are scored in such a way that higher scores represent the presence of a larger degree of job demands or job resources. To achieve this, the scores for several items (items 5, 9, 20 and 21) are reversed prior to calculation of total scores.

Items and information about them are presented in Table S1.

Table S1. Item descriptions, Job Demands and Resources Questionnaire

Item		Category	Sub-category	Scoring	Developed from
1.	My achievements are recognised by my supervisor / manager	Job Resources	Rewards and recognition	Standard	[1]
2.	The job requires me to keep track of more than one thing at a time	Job Demands	Cognitive demands	Standard	[1]
3.	My work requires a lot of concentration	Job Demands	Cognitive demands	Standard	[2]
4.	The job gives me a chance to use my personal initiative or judgment in carrying out the work	Job Resources	Job control	Standard	[1]
5.	Only the management decides what everybody has to do	Job Resources	Participation	Reversed	[3]
6.	My work is emotionally demanding	Job Demands	Emotional demands	Standard	[2]
7.	My supervisor is concerned about the welfare of the people that work for him/her	Job Resources	Supervisor support	Standard	[1]
8.	The job gives me considerable opportunity for independence and freedom in how I do the work	Job Resources	Job control	Standard	[1]

Item		Category	Sub-category	Scoring	Developed from
9.	I always have enough time to perform my tasks	Job Demands	Time pressure	Reversed	[3]
10.	I receive feedback on my performance from other people in my organization (such as my manager or coworkers)	Job Resources	Feedback	Standard	[1]
11.	I get enough feedback about the quality of my performance	Job Resources	Feedback	Standard	[3]
12.	My supervisor / manager inspires me to do my best work	Job Resources	Supervisor support	Standard	[4]
13.	My colleagues are willing to give me help if I ask for it	Job Resources	Social support	Standard	[4]
14.	My job is physically taxing	Job Demands	Physical workload	Standard	[3]
15.	It is physically taxing for me to get used to my working times	Job Demands	Shift work	Standard	[3]
16.	My achievements are recognised by my colleagues	Job Resources	Rewards and recognition	Standard	[1]
17.	My performance is rewarded properly	Job Resources	Rewards and recognition	Standard	[3]

Item		Category	Sub-category	Scoring	Developed from
18.	My supervisor uses his / her influence to help me solve problems	Job Resources	Supervisor support	Standard	[1]
19.	The job allows me to make my own decisions about how to schedule my work	Job Resources	Job control	Standard	[1]
20.	My working hours allow me to meet my family and personal obligations	Job Demands	Work-home interference	Reverse	[2]
21.	My physical working conditions – climate, light, noise, design of the working place, and material – are alright	Job Demands	Physical environment	Reverse	[3]
22.	My job requires working very hard or very fast	Job Demands	Workload	Standard	[2]
23.	I feel my job is secure	Job Resources	Job security	Standard	[3]
24.	My contact with persons to whom I have to offer services is demanding	Job Demands	Recipient contact demands	Standard	[3]

Item		Category	Sub-category	Scoring	Developed from
25.	I have good relationships with my colleagues	Job Resources	Social support	Standard	[2]

REFERENCES

1. Morgeson FP, Humphrey SE: The Work Design Questionnaire (WDQ): Developing and validating a comprehensive measure for assessing job design and the nature of work. J Appl Psychol 2006, 91:1321-1339; doi:10.1037/0021-9010.91.6.1321.
2. Van den Broeck A, De Cuyper N, De Witte H, Vansteenkiste M: Not all job demands are equal: Differentiating job hindrances and job challenges in the Job Demands-Resources model. European Journal of Work and Organizational Psychology 2010, 19:735-759; doi:10.1080/13594320903223839.
3. Demerouti E, Bakker AB, Nachreiner F, Schaufeli WB: The Job Demands-Resources Model of burnout. J Appl Psychol 2001, 86:499-512; doi:10.1037//0021-9010.86.3.499.
4. Bakker AB, Demerouti E, Euwema MC: Job resources buffer the impact of job demands on burnout. J Occup Health Psychol 2005, 10:170-180; doi:10.1002/hrm.20004.
